# The Effect of Octapeptide Repeats on Prion Folding and Misfolding

**DOI:** 10.3390/ijms22041800

**Published:** 2021-02-11

**Authors:** Kun-Hua Yu, Mei-Yu Huang, Yi-Ru Lee, Yu-Kie Lin, Hau-Ren Chen, Cheng-I Lee

**Affiliations:** Department of Biomedical Sciences, National Chung Cheng University, 168 University Road, Min-Hsiung Chia-Yi 62102, Taiwan; ykhuna@gmail.com (K.-H.Y.); hb07925@hotmail.com (M.-Y.H.); sane11989@hotmail.com (Y.-R.L.); kie82123@gmail.com (Y.-K.L.); biohrc@ccu.edu.tw (H.-R.C.)

**Keywords:** prion, octapeptide, folding, misfolding, fibril

## Abstract

Misfolding of prion protein (PrP) into amyloid aggregates is the central feature of prion diseases. PrP has an amyloidogenic C-terminal domain with three α-helices and a flexible tail in the N-terminal domain in which multiple octapeptide repeats are present in most mammals. The role of the octapeptides in prion diseases has previously been underestimated because the octapeptides are not located in the amyloidogenic domain. Correlation between the number of octapeptide repeats and age of onset suggests the critical role of octapeptide repeats in prion diseases. In this study, we have investigated four PrP variants without any octapeptides and with 1, 5 and 8 octapeptide repeats. From the comparison of the protein structure and the thermal stability of these proteins, as well as the characterization of amyloids converted from these PrP variants, we found that octapeptide repeats affect both folding and misfolding of PrP creating amyloid fibrils with distinct structures. Deletion of octapeptides forms fewer twisted fibrils and weakens the cytotoxicity. Insertion of octapeptides enhances the formation of typical silk-like fibrils but it does not increase the cytotoxicity. There might be some threshold effect and increasing the number of peptides beyond a certain limit has no further effect on the cell viability, though the reasons are unclear at this stage. Overall, the results of this study elucidate the molecular mechanism of octapeptides at the onset of prion diseases.

## 1. Introduction

Transmissible spongiform encephalopathies (TSE or prion diseases) are fatal neurodegenerative disorders with sporadic, infectious and inherited etiologies. The prion proteins (PrP) play an essential role in the pathogenesis of prion diseases. According to the protein-only hypothesis [1], the normal cellular isoform of the prion protein (PrP^C^) misfolds and aggregates into a disease-associated state termed PrP^Sc^ (Sc stands for Scrapie) [2]. PrP^C^ is sensitive to protease K (PK)-digestion, while PrP^Sc^ is resistant to PK-digestion. PrP^C^ is ubiquitous throughout the central nervous system of mammals [3].

Misfolding of PrP^C^ into PrP^Sc^ readily causes various types of transmissible spongiform encephalopathies in animals, such as Creutzfeldt-Jakob disease (CJD) in humans, bovine spongiform encephalopathy (BSE) in cattle and Scrapie in sheep. Approximately 15% of CJD are genetic disorders [4]. Conventional single point mutations and multiple insertion mutations in an octapeptide region in the N-terminal domain contribute approximately equally [5]. Deletion in the octapeptide region of the N-terminal domain occurs in approximately 2% of the Caucasian population [6].

Nuclear magnetic resonance (NMR) studies indicate that PrP^C^ possesses a large α-helical domain and a short two-stranded anti-parallel β-sheet in the C-terminal domain (residues 121–231 inclusive) and a flexible N-terminal domain (residues 23–120 inclusive) [7]. In contrast, PrP^Sc^ is rich with cross β-sheets, yet few α-helical structures [8]. The C-terminal domain of PrP has been considered an amyloidogenic region because amyloid fibrils can be converted from PrP_121–231_ (equivalent to mouse PrP_23–230_) proteins, which lack the entire unstructured N-terminal domain of the protein [9]. The N-terminal domain of mouse PrP contains five tandem repeats of the octapeptide P(H/Q)GG(-/G/S/T)WGQ sequence including a leading octapeptide PQGGTWGQ from residue 51 to residue 58 (inclusive) and the following four octapeptide repeats PHGG(G/S)WGQ from residue 59 to residue 90 (inclusive). These four octapeptide repeats bind to divalent cation including Cu^2+^, Mn^2+^ or Zn^2+^ due to the chelating effect of histidine residues. To be noted, the leading peptide in humans is PQGGGTWGQ nonapeptide without histidine to bind Cu^2+^. Thus, the repeat number of these octapeptides has been considered as four in some studies [10,11].

Across species, the octapeptide region is among the represented portions of the PrP sequence [12], suggesting that this region plays a critical role in PrP^C^ function. The octapeptides are structurally flexible and are outside of the amyloidogenic C-terminal domain. This octapeptide region in prion diseases has been greatly underestimated. However, a survey on CJD patients suggested a correlation between the number of octapeptide repeats and the age of onset; when the number of octapeptide repeats is decreased, the onset of CJD is postponed [13], whereas a large number of octapeptide insertions induces large plaques and early onset [14]. In octapeptide deleted PrP, CJD syndrome is not well recognized [4]. In summary, the number of octapeptide repeats is critical in prion diseases and we need to understand how the variation of octapeptide repeats modulates the mechanism of the diseases.

**Figure 1:**
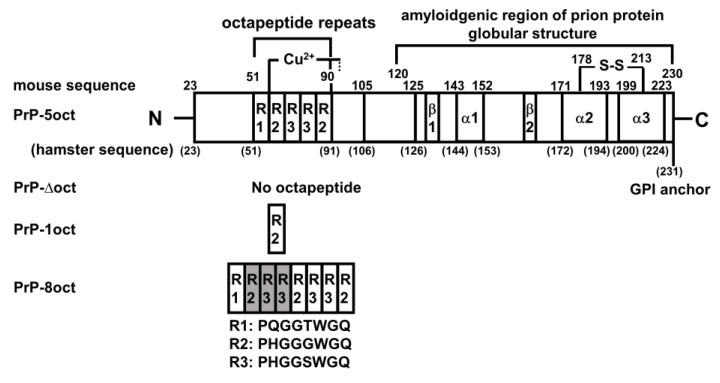
Representative scheme of prion sequence and prion proteins (PrP) variants with different number of octapeptide repeats. The sequences of the octapeptide repeats are represented by R1, R2 and R3. Three α-helices are labeled as α1, α2 and α3. Two β-strands are labeled as β1 and β2.

As the number of octapeptide repeats is critical in the pathology of prion diseases, we have worked on full-length mouse PrP carrying 5 repeats of octapeptides (PrP-5oct) without any peptide tag. Its deletion/insertion resulted in three other PrP variants: PrP without any octapeptide (PrP-Δoct), PrP with one (PrP-1oct) and eight octapeptide repeats (PrP-8oct) as illustrated in Figure 1. We have analyzed the protein structure, the structural stability of these protein variants and the kinetics of their amyloid conversion as well as structural characteristics of the amyloid fibrils. The cytotoxicity of these four PrP variants is also examined at the cellular level.

## 2. Results

### 2.1. The Number of Octapeptide Repeats Affects the Structure and the Thermal Stability of Prion Proteins

**Figure 2:**
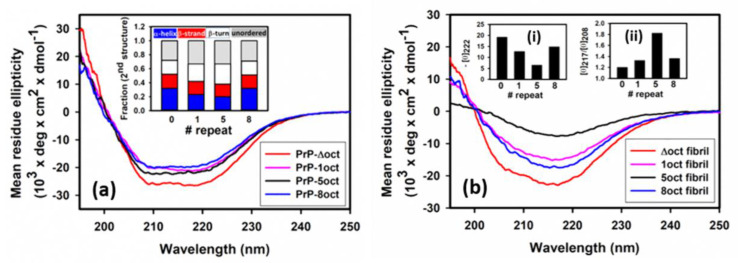
Circular dichroism (CD) analysis of (**a**) PrP variants and (**b**) fibrils converted from four PrP variants.

**Figure 3:**
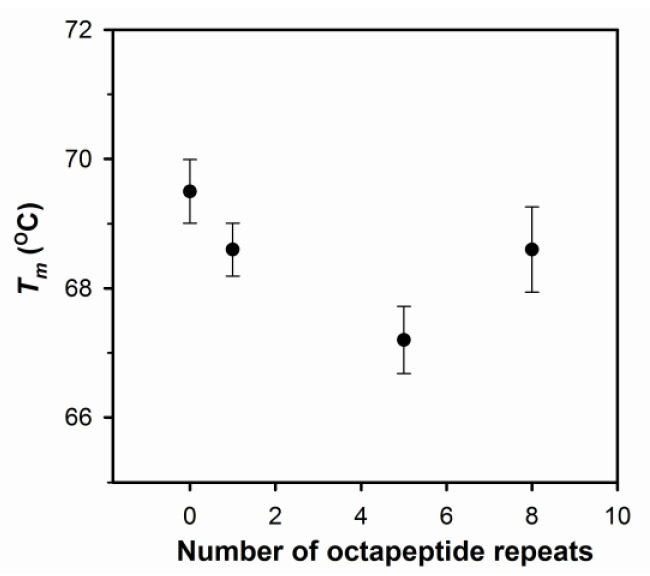
Thermal stability of four PrP variants compared by their T*_m_* values.

According to the NMR studies, PrP is rich with α-helices located in the C-terminal domain, while the N-terminal domain is unstructured [7]. Therefore, in the determination of mean residue ellipticity, only the residues in the C-terminal domain (residues 120 to 230) are counted. As shown in Figure 2a, the circular dichroism (CD) spectra of all PrP variants illustrate high amounts of α-helical structures found at negative peaks of 208 nm and 222 nm. The analysis of the fraction of the secondary structure is shown in the insert of Figure 2a. All PrP variants have the same fraction of β-sheets. In comparison to PrP-5oct, both deletion and insertion of octapeptides increase the fraction of α-helical structure. The observation that the change of α-helical structures are related to the number of octapeptides indicates that octapeptides in the N-terminal domain can effectively affect the α-helices dominant in the C-terminal domain of PrP. In addition, thermal stability of α-helices in PrP variants measured by their melting temperature (T*_m_*) values is compared in Figure 3. An increase in T*_m_* was observed in both deletion and insertion of octapeptides. In other words, PrP-5oct has the least thermal stability. The correlation of T*_m_* is very similar to the number of octapeptide repeats vs. the fraction of α-helical structure (insert in Figure 2a).

### 2.2. The Expansion Rather Than the Deletion of Octapeptides Enhances the Kinetics of Fibril Conversion

**Figure 4:**
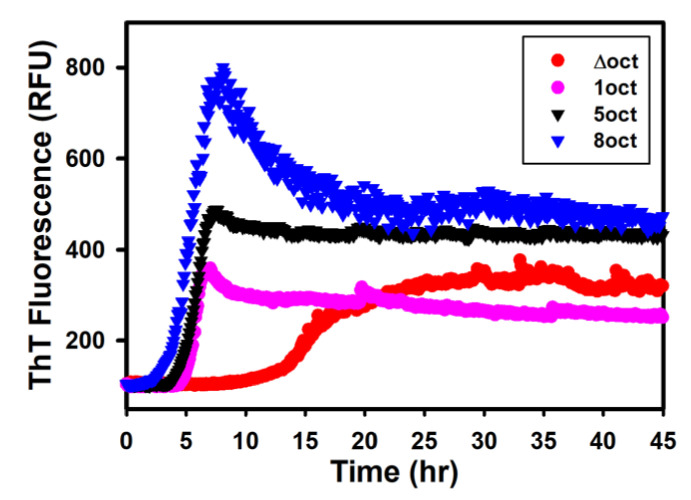
Kinetics of fibril conversion from four PrP variants.

Some selected data points in the kinetics of the fibril conversion are shown in Figure 4 and analyzed with a nucleation dependent polymerization model. Among four tested variants, PrP-8oct has the shortest lag time of 3.9 h, while the PrP-1oct and PrP-5oct have their lag time of 5.0 and 4.8 h, respectively. PrP-Δoct has the longest lag time of 11.5 h. If measuring the quantity of amyloid fibrils by maximum ThT fluorescence, converted fibrils are significantly higher in PrP-8oct and lower in PrP-Δoct and PrP-1oct, in comparison to PrP-5oct.

### 2.3. The Number of Octapeptide Repeats Affects the Structures of Fibrils

**Figure 5:**
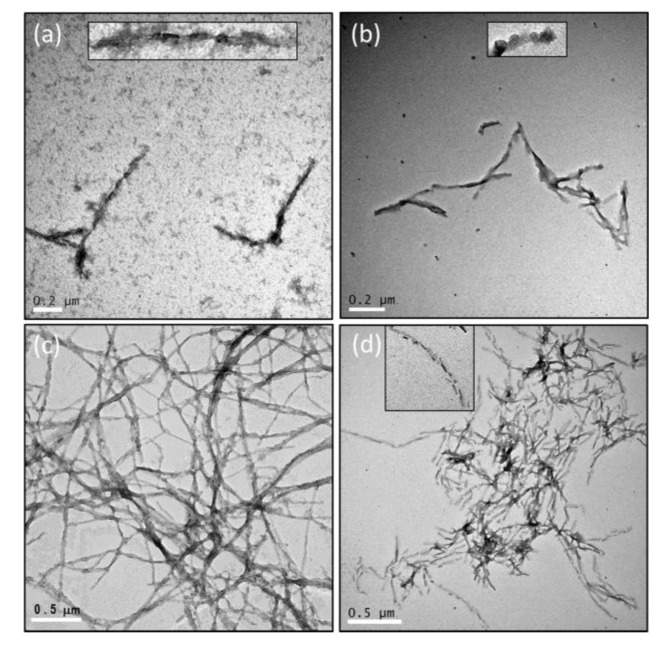
Transmission electron microscopy (TEM) images of fibrils converted from four PrP variants (**a**) PrP-Δoct, (**b**) PrP-1oct, (**c**) PrP-5oct and (**d**) PrP-8oct.

The fibrils converted from PrP variants were analyzed for their secondary structure by CD spectroscopy (Figure 2b) and imaged with transmission electron microscopy (TEM) (Figure 5). After deletion or insertion of octapeptides, the fibrils have structures different from PrP-5oct. During the fibril conversion, PrP changes the structure from α-helical structure into β-sheets but the level of the structural conversion is quite different among the four PrP variants. The levels of α-helical structure represented by the negative peaks at 222 nm in four PrP variants were compared as shown in the insert (i). PrP-5oct preserves the least amount of α-helical structure. Furthermore, to determine the contents of the structural change in each condition, the negative peaks representing β-sheet and α-helical structures at 217 nm and 208 nm, respectively, were put into ratios to characterize the helix-to-sheet structural change in fibril conversion. As shown in the inset (ii) of Figure 2b, PrP-5oct has the highest value of [θ]217/[θ]208 at 1.83 and PrP-8oct has a value of 1.36. In PrP-Δoct and PrP-1oct, the values of [θ]217/[θ]208 are only 1.20 and 1.32, respectively. The correlation of these ratios is similar to the number of octapeptide repeats vs. T*_m_* (see Figure 3 and inset (ii) of Figure 2b). In other words, PrP-5oct has the weakest thermal stability and has the greatest structural change during fibril conversion.

As illustrated in Figure 5, PrP-5oct significantly turns into long silk-like fibrils and the fibrils populate evenly over the field. A high number of fibrils are observed in PrP-8oct as well. However, PrP-8oct fibrils are extremely fragmented along the long axis of their fibrils as compared to PrP-5oct fibrils. In addition, PrP-8oct fibrils aggregate, consistent with the decrease of ThT fluorescence after reaching the maximum reading in Figure 4. No oligomers are observed in the PrP-5oct and PrP-8oct fibril samples. In contrast to the long fibrils in PrP-5oct and PrP-8oct, PrP-Δoct has very few fibrils formed and has many loosely formed oligomers present. The PrP-1oct has very few mature fibrils but retains moderate ThT-fluorescence as shown in Figure 4. Similar to PrP-Δoct, some protofibrils are present in the PrP-1oct fibril sample and these protofibrils seem to be more well-formed then those oligomers in PrP-Δoct. Among the limited amount of PrP-Δoct and PrP-1oct fibrils, a twisted form of fibrils is also identified. This twisted form is highly distinguishable from the silk-like form of PrP-5oct fibrils and the fragmented form of PrP-8oct fibrils. Overall, the population of these four fibril samples correlates with the observation that increasing the number of octapeptide repeats causes accumulation of large plaques [14].

A previous study on the correlation between structural stability and the incubation period indicated that less stable prions replicate more rapidly than stable prions and that the less stable prions quickly replicate during a fast breakup [15]. As aforementioned, PrP-5oct has the least stable structure, thus, it has the highest yield of fibril formation. The formation of fibril seeds is difficult when the fibrils cannot be easily fragmented, such as the twisted fibrils of PrP-Δoct and PrP-1oct and typical silk-like fibrils of PrP-5oct as observed in many other amyloid proteins [16]. Considering the weak α-helical structure and fragmented fibrils observed in PrP-8oct, it is highly possible that PrP-8oct is highly flexible to carry out structural change to form amyloid fibrils. In addition, these PrP-8oct fibrils easily fragment to serve as seeds for elongation of other misfolded PrP-8oct monomers. With abundant fibril seeds, PrP-8oct starts elongation in a short time. The density of fibrils shown in TEM images is very high in PrP-5oct and PrP-8oct but is very low in PrP-Δoct and PrP-1oct. The ThT-fluorescence readings of PrP-Δoct and PrP-1oct fibrils are approximately half of the ThT-fluorescence reading of PrP-5oct fibrils. The major reason is that the fibrils with different structures carry different levels of ThT-fluorescence [17]. The fibrils cannot be precisely quantified simply based on ThT-fluorescence of the four PrP variants because fibrils converted from these four PrP variants have different misfolding pathways resulting in distinguishable structures.

**Figure 6:**
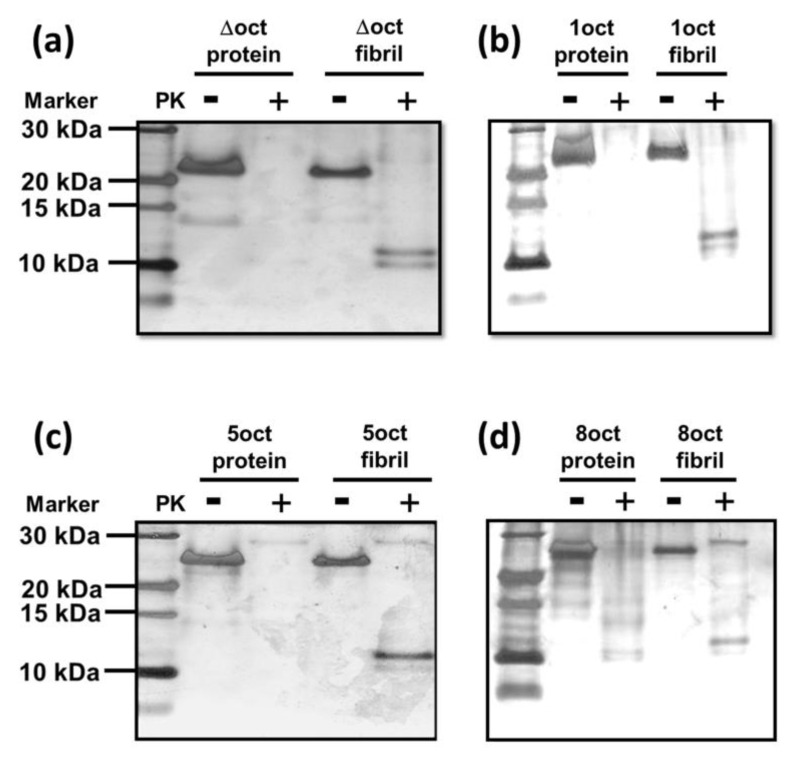
PK-cleavage (PK: PrP = 1:50) of PrP variants (**a**) PrP-Δoct, (**b**) PrP-1oct, (**c**) PrP-5oct and (**d**) PrP-8oct in their protein and fibril forms analyzed with tricine-sodium dodecyl sulfate-polyacrylamide gel electrophoresis (SDS-PAGE).

PK-resistance is a unique property of PrP^Sc^ and a PK-digestion assay is one of the best ways to distinguish PrP^C^ and PrP^Sc^. Thus, we conducted PK-digestion assays on four PrP variants in both protein and fibril forms as illustrated in Figure 6. In their protein forms, PrP-Δoct, PrP-1oct and PrP-5oct are all PK-sensitive, while PrP-8oct (25 kDa) is partially resistant to PK as shown by the residual PrP band and small protein bands between 10 and 15 kDa. The PK-resistant protein fragments in PrP-8oct are critical as these octapeptide insertions are toxic before fibril conversion is carried out. In their fibril forms, all PrP variants have PK-resistant bands observed at ~10 kDa, regardless of the structures of these fibrils. Consistent with a study of cell-expressed hamster PrP, PK-resistance is significantly increased upon octapeptide insertion [18].

### 2.4. The Number of Octapeptide Repeats Affects the Cytotoxicity of the Amyloid Fibrils

**Figure 7:**
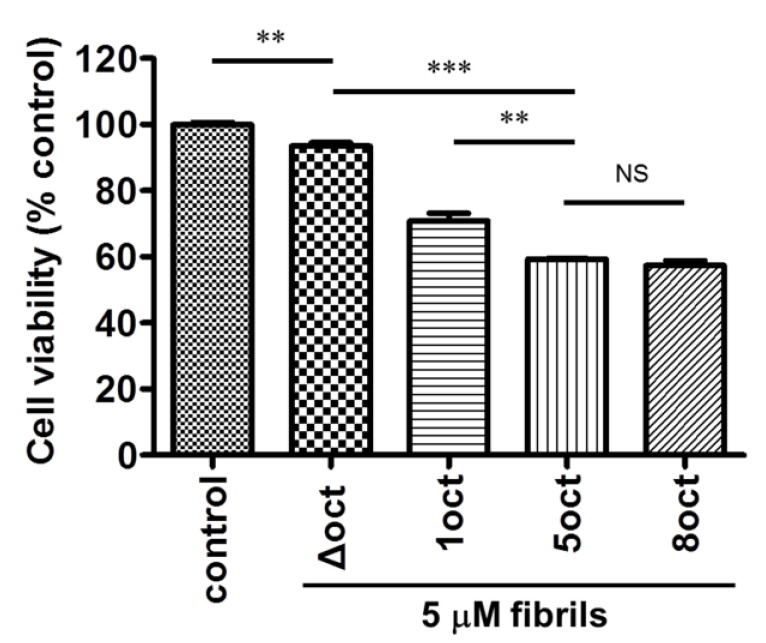
Cell viability of N2a cells upon treatment with fibrils converted from four PrP variants. The control sample is N2a cells without fibril treatment. **: *p* < 0.01, ***: *p* < 0.001, NS: non-significant.

After the characterization of the amyloid fibrils in a cell-free system, we further examined the toxicity of these fibrils in neuron-like differentiated mouse neuroblastoma (N2a) cells by cell viability assay as shown in Figure 7. In comparison to the untreated N2a cells, cell viability decreases with the number of octapeptide repeats of the fibril samples. Fibrils converted from PrP with a smaller number of octapeptide repeats contribute lower cytotoxicity but insertion of octapeptides (PrP-8oct) does not increase cytotoxicity in comparison to PrP-5oct. In our PrP-Δoct fibril sample, lots of loosely formed oligomers are observed but they affect cell viability weakly. PrP-1oct fibrils have similar structures to PrP-Δoct fibrils but PrP-1oct fibrils are more cytotoxic than PrP-Δoct fibrils. This cytotoxicity difference could be due to the presence of protofibrils rather than oligomers in the PrP-1oct fibril sample. PrP-5oct and PrP-8oct fibrils have distinct structures but their cytotoxicity is independent of their fibril structures, although the fragmentation of PrP-8oct fibrils produce some short fibrils. There might be some threshold effect and increasing the number of peptides beyond a certain limit has no further effect on the cell viability, though the reasons are unclear at this stage. Considering that PrP-8oct converts into fibrils quickly as it causes damage earlier than PrP-5oct does, it is correlated with the early onset of diseases observed clinically [13]. There are fewer cases of patients with prion diseases [14] and weak cytotoxicity of PrP-Δoct and PrP-1oct correlates with fewer cases of patients with prion diseases.

## 3. Discussion

Extensive studies of recombinant prion proteins indicate that PrP_90–231_ can potentially convert to amyloid fibrils with PK-resistance [19] and are encephalopathological to mice [20]. Extending the N-terminal sequence to full-length PrP, PrP_23–231_ tends to produce similar amyloid fibrils with amyloidogenic cores in the C-terminal domain [21]. An amyloidogenic core has been determined to be PrP_121–231_, when fibrillization of PrP_121–231_ was reported [9]. The octapeptides are not located in the amyloidogenic core, thus, the critical role of octapeptides in the flexible N-terminal domain has been strongly underestimated.

Our CD spectra indicate that insertion and deletion of octapeptides change the fraction of α-helical structure. This result indicates that the N-terminal domain has some influence over the globular structure in the C-terminal domain. Our CD result is consistent with a previous study that showed truncation of the N-terminal domain causes a shift in the NMR spectrum [22]. The N-terminal domain has an extremely high level of disorder and this property decreases significantly when extending the protein sequence to the globular C-terminal domain. Helix I (equal to α1 in Figure 1) has been considered a highly mobile segment from the amyloid core [23,24]. Changing the number of octapeptide repeats initiates the weakening of the helical structures and this flexibility promotes its role as a hydrophilic seed in prion aggregates [25]. As a result, PrP with multiple octapeptide repeats has a higher tendency to weaken the rigid α-helical structure when carrying out structural conversion. In contrast, PrP with a lower number of octapeptide repeats tends not to weaken α-helical structure as much. Therefore, PrP-5oct and PrP-8oct have similar structures, whereas PrP-Δoct and PrP-1oct have distinct structural differences from PrP-5oct.

Two types of α-synuclein fibrils converted under identical conditions have been identified [26]. An animal study of α-synuclein fibrils with distinct structure indicated that structure might determine the toxicity of the fibrils [27]. Similarly, two distinct structures of PrP fibrils converted from the same protein produced different levels of cytotoxicity [28]. Huntington’s disease has also been ascribed to misfolding of huntingtin protein caused by an increase of polyglutamine [29]. Similarly, insertion of octapeptides is correlated with the early onset of CJD [13,14]. Our study indicates that insertion of octapeptides weakens the α-helical structure, accelerates prion fibrillation and causes significant changes in fibril structure. The deletion of octapeptides strengthens the α-helical structure. PrP variants with different structures then misfold into different amyloid structures. Immunocytochemical examination on patients indicated that the number of octapeptide repeats determines the type of amyloid deposit in the cerebellum and the corresponding prion encephalopathy [30]. As the prion proteins carrying different numbers of octapeptide repeats convert to distinct structures of fibrils, these fibrils could possibly deposit in different parts of the cerebellum and reflect the severity of the diseases.

## 4. Materials and Methods

### 4.1. Plasmid Preparations of PrP-Δoct, PrP-1oct and PrP-8oct

Two oligonucleotides for PrP-∆oct were used in this reaction including a forward primer (5′-TATCCCGGGCAGGGAAGCCCTGGAGGCAACCGTTACCCA-3′) and a reverse primer (5′-TGGGTACCCCCTCCTGGGTAACGGTTGCCTCCAG-3′). In a template-repeated polymerase chain reaction (TR-PCR) [31], the two primers were also used as templates.

Similarly, PrP-1oct plasmid was prepared based on TR-PCR from the following primers: a forward primer (5′-TATCCCGGGCAGGGAAGCCCTGGAGGCAAC CGTTACCCACCCCACGGTGG-3′) and a reverse primer (5′-ATGGGTACCC CCTCCTTGTCCCCAGCCACCACCGTGGGGTGGGTAA-3′).

The wild type mouse PrP (PrP-5oct) gene has been constructed previously [32]. To delete five octapeptide repeats in PrP-5oct, a SmaI and a KpnI cleavage sites were used to remove all octapeptides in mouse PrP-5oct. After the digestion with restriction enzymes, the TR-PCR products were subcloned into pET101/D-TOPO vectors. To insert additional copies of the octapeptide into PrP-5oct, we first conducted site-directed mutagenesis to change the sequence at codons 54 and 55 of PrP-5oct to create a BamHI cleavage site. After digestion with restriction enzymes Bsu36I and BamHI, additional oligonucleotides for insertion of three octapeptide repeats were subcloned into PrP-5oct resulting in PrP-8oct. The inserted oligonucleotides are as following. The forward primer: TCAGGGTGGCACCTGGGGGCAGCCCCACGGTGGTGGCTGGGGACAACCACATGGTGGTTCTTGGGGTCAACCTCACGGTG. The reverse primer: GATCCACCGTGAGGTTGACCCCAAGAACCACCATGTGGTTGTCCCCAGCCACCACCGTGGGGCTGCCCCCAGGTGCCACCC. To be noted, this engineering introduces a Thr to Ser change at codon 55 in PrP-5oct but this Ser becomes part of R3 that follows the inserted sequence as shown in Figure 1. Therefore, except for the insertion, the wild type sequence is not changed.

### 4.2. Expression and Purification of PrP-Δoct, PrP-1oct, PrP-5oct and PrP-8oct

The plasmids pET101 encoding PrP-Δoct, PrP-1oct, PrP-5oct and PrP-8oct were transformed into competent *Escherichia coli* BL21 star™ (DE3) (Life Technologies, Grand Island, NY, USA) cells, in 50 mL of LB medium for approximately 16 hours. From a large-scale culture, cells were transferred to a TB medium and grown until an optical density at 600 nm reached 0.6. Subsequent addition of 1 mM isopropyl β-D-1-thiogalactopyranoside induced expression of proteins. The PrP-5oct and PrP-8oct proteins were purified by immobilized metal affinity chromatography and reversed-phase C4-HPLC as modified from previously reported procedures [32,33,34]. The PrP-Δoct and PrP-1oct proteins were purified by cation exchange chromatography with SP Sepharose and reversed-phase C4-HPLC as previously described [35]. The purified PrP was confirmed by sodium dodecyl sulfate-polyacrylamide gel electrophoresis (SDS-PAGE) and electrospray ionization-mass spectrometry to be a single population with the correct molecular weight.

### 4.3. Circular Dichroism Spectroscopy (CD)

CD spectra of mouse PrP variants were recorded with a spectrometer (Jasco J-815, Tokyo, Japan). For measurements in the far-UV region, a quartz cell with a path length of 0.1 cm was used in a nitrogen atmosphere. For protein samples, the concentration was kept constant at 10 μM in 10 mM Tris (pH 7.3). For fibril samples, the samples were dialyzed against water prior to the measurements. A set of five scans, with a scan speed of 50 nm per minute, was performed at 20 °C. The fraction of the secondary structures was analyzed by DichroWeb [36,37].

The heat-induced denaturation of proteins was conducted with heating protein solutions at the rate of 1 °C/min and the ellipticity at 222 nm, was recorded every 0.5 °C. The thermal stability of proteins was analyzed based on the Gibbs-Helmholtz equation and the melting temperatures (T*_m_*) of mouse PrP variants were determined.

### 4.4. Kinetics of Fibril Conversion from PrP Variants

For fibril conversion, four PrP proteins were added into 50 mM MES (pH 6) in the presence of 2 M guanidine hydrochloride (GdnHCl). The samples were incubated at 37 °C with vigorous shaking, as described in previous studies on prion fibrillation [34,35,38]. At the end of the experiment, the fibril samples were dialyzed against water for further analysis. For the kinetics experiment, the fibril conversion was carried out in a multiwell plate rotating at 600 rpm at 37 °C monitored by the fluorescence of thioflavin T (ThT) in a microplate reader (FLUOstar Omega, BMG LABTECH, Ortenberg, Germany). The kinetics of fibril conversion were analyzed by the nucleation dependent polymerization model [22]. The lag phase of amyloid formation from PrP can be determined by fitting the time-dependent changes in the ThT fluorescence (F) over time (t) of the reaction as expressed in the following equation, F = F_0_ + ΔF/(1 + exp[*k*(*t_m_* –*t*)]). Where F_0_ is the minimum level of ThT fluorescence during the lag phase, ΔF is the difference of ThT fluorescence between the maximum level (steady state) and the minimum level (lag phase), *k* is the fibril growth rate (h^−1^) and *t_m_* is the observed time at the midpoint of transition. The lag time (*t_l_*) of fibril formation can be calculated as: *t_l_* = *t_m_* − 2/*k*.

### 4.5. Transmissible Electron Microscopy (TEM)

The diluted fibril samples were adsorbed onto carbon-coated 200-mesh copper grids for 30 s and washed twice with PBS buffer. Subsequently, the adsorbed fibrils were stained with 2% tungsten phosphoric acid on copper grids and then washed with H_2_O. The samples were air-dried before imaging. The TEM images were collected using a TEM (Hitachi H-7100, Tokyo, Japan).

### 4.6. PK-Digestion Assay

In this assay, 10 μM of protein and fibril samples were treated with PK (29 kDa) at a PrP-to-PK ratio of 1:50 at 37 °C in 100 mM Tris (pH 7.5) for one hour. After PK-digestion, samples were analyzed by tricine-SDS-PAGE [39].

### 4.7. Cell Culture and Viability Assay

N2a cells were maintained in Dulbecco’s modified Eagle’s medium (DMEM) supplemented with penicillin/streptomycin and 10% (*v/v*) fetal bovine serum in a humidified atmosphere with 5% CO_2_ at 37 °C. In the following incubation, N2a cells (1 × 10^4^) were seeded to 96-well plates for 24 h in a serum-free DMEM medium to differentiate into a neuron-like morphology and then treated with PrP fibrils. After 72 h of incubation, the medium was removed and the cell plates were washed by PBS. Subsequently, 10% (*v/v*) WST-1 was added into the plates and incubated with cells for 3 h in the dark. During the incubation, the cleavage of the tetrazolium salt WST-1 to formazan by cellular mitochondrial dehydrogenases was carried out. The cell viability was determined by the absorption of formazan recorded at 450 nm using an ELISA reader. The collected data were compared using *T*-test statistical analysis.

## 5. Conclusions

Increasing octapeptide repeats accelerates prion fibrillation and induces significant change in fibril structure, whereas decreasing octapeptide repeats enhances the intramolecular α-helical structures but forms fewer twisted fibrils. Increasing the number of octapeptide repeats turns PrP into partially PK-resistant before fibril conversion. Deletion of octapeptides weakens the cytotoxicity but insertion of octapeptides does not increase the cytotoxicity. The effect of octapeptide repeats observed in this study explains the molecular mechanism of octapeptide repeats at the onset of prion diseases. Prior to this research, the octapeptides were not considered as important in prion diseases. Our study indicates that the octapeptides are critical in folding and misfolding of prion proteins.

## Data Availability

Not applicable.
